# *Bifidobacterium breve* PRL2020: Antibiotic-Resistant Profile and Genomic Detection of Antibiotic Resistance Determinants

**DOI:** 10.3390/microorganisms11071649

**Published:** 2023-06-24

**Authors:** Francesco Di Pierro, Ilenia Campedelli, Patrick De Marta, Fabio Fracchetti, Antonio Del Casale, Ilaria Cavecchia, Mariarosaria Matera, Massimiliano Cazzaniga, Alexander Bertuccioli, Luigina Guasti, Nicola Zerbinati

**Affiliations:** 1 Scientific & Research Department, Velleja Research, 20125 Milan, Italy; 2 Department of Medicine and Surgery, University of Insubria, 21100 Varese, Italy; 3 Microbion, San Giovanni Lupatoto, 37057 Verona, Italy; 4 Microbiomic Department, Koelliker Hospital, 10134 Turin, Italy; 5 Department of Pediatric Emergencies, Misericordia Hospital, 58100 Grosseto, Italy; 6 Department of Biomolecular Sciences, University of Urbino Carlo Bo, 61122 Urbino, Italy

**Keywords:** LMG S-32458, amoxicillin, clavulanic acid, erythromycin, rifaximin, ampicillin, microbiota, probiotics

## Abstract

Antibiotics are one of the greatest scientific achievements of modern medicine, but excessive use is creating challenges for the future of medicine. Antibiotic resistance (AR) is thought to cause changes in bowel habits and an increased risk of gastroenteritis, but it may also increase the risk of overweight, obesity, autoimmune and atopic diseases, and a low response to vaccines and cancer, likely mediated by antibiotic-induced gut dysbiosis. Probiotic add-on therapy could partially prevent antibiotic-induced gut dysbiosis, but their antibiotic sensitivity features likely limits this potential. The EFSA (European Food Safety Authority) guidelines consider the use of probiotics whose antibiotic-resistant profile could be transferable an important hazard. Recently, a strain of *B. breve* (PRL2020) has shown to be resistant to amoxicillin and amoxicillin-clavulanate (AC) by applying the microdilution protocol according EFSA guidelines. After verifying that horizontal gene transfer is unlikely to take place, this feature suggests its concomitant use with these specific antibiotics. The results of our tests demonstrated that the strain PRL2020 is indeed endowed with amoxicillin- and AC-resistant properties and that it is also insensitive to ampicillin. In-depth analysis of the annotated genome sequence of *B. breve* PRL2020 was employed to query the Comprehensive Antibiotic Resistance Database (CARD) using Resistance Gene Identifier (RGI) software (version 5.2.1). The similarity among the AR determinants found was studied through nucleotide sequence alignment, and it was possible to verify not only the absence of genes explaining these features in the flanking regions but also the presence of genetic sequences (*rpoB* and *erm(X)*) putatively responsible for rifampicin and erythromycin resistance. Both features are not phenotypically expressed, and for these antibiotics, the strain is within the EFSA limits. Analysis of the flanking regions of these genes revealed possible mobile elements upstream and downstream only in the case of the *erm(X)* gene, but the features of the Insertion Sequences (IS) are described as not to cause horizontal transfer. Our findings on strain PRL2020 demonstrate that its AR profile is compatible with antibiotics when taken with the aim of reducing the risk of dysbiosis.

## 1. Introduction

Antibiotics are generally considered one of the greatest scientific achievements of the 20th century and, together with vaccines, have the potential to extend life by up to 30 years for those who can access them. However, their use has increased in recent years, probably beyond necessity. For example, between 2000 and 2015, worldwide antibiotic use increased by about 65% [1]. The flip side of this increase is the well-known phenomenon of antibiotic resistance (AR). Forecasts predict that AR may be the greatest challenge facing future medicine. According to the review on antimicrobial resistance commissioned in 2016 by UK healthy authorities, in 2050, tetanus will likely kill 60,000 people worldwide per year; cholera about 120,000; measles about 130,000; road accidents around 1,200,000; diarrhoea about 1,400,000; diabetes about 1,500,000; cancer about 8,200,000, antibiotic resistance around 10,000,000 [2]. The pathogens that most are thought to pose a global threat to humans are *Enterococcus faecium*, *Staphylococcus aureus*, *Klebsiella pneumoniae*, *Acinetobacter baumannii*, *Pseudomonas aeruginosa*, and *Enterobacter* spp. [3]. The spread of antibiotic resistance is mainly due to horizontal gene transfer among bacterial cells, and this phenomenon is clearly amplified by antibiotic exposure [4]. In fact, the administration of antibiotics to healthy volunteers increases the percentage of bacterial cells resistant to the administered antibiotics by more than 50% in less than four days. This antibiotic-resistant profile lasts more than 42 days following the last administration and is still not completely extinguished after 180 days [5]. A common side-effect is changes in bowel habits, which occurs in up to 30% of individuals treated by antimicrobial agents. Most cases are benign and simply due to a transient dysbiosis [6]. In some cases, however, the antibiotic-induced alteration of the gut microbiota leads to the establishment of pathogens, of which *Clostridium difficile* is likely the most important [7] but not the only one (as demonstrated by the incidence of post-antibiotic *Clostridium perfringens* induced diarrhoea) [8]. Moreover, an increasing number of studies seem to correlate the increasing diffusion of antibiotics with an increased risk, especially but not restricted to children, of pathological manifestations, such as overweight and obesity, autoimmune diseases, atopic diseases, low response to vaccines and even cancer [9,10,11,12,13,14,15]. It is believed that at the basis of these events, there may be an alteration of the consortium structure of the colon microbiota that is possibly characterized by (i) a slight increase in the bacterial load, (ii) the overgrowth of Gram-negative bacterial species, (iii) the reduction in microbiota richness and, from a taxonomic perspective, (iv) the frequently reduction in both the butyrate-producers of the phylum Firmicutes and the species belonging to the *Bifidobacterium* genus, along with the increase in the relative percentage of species belonging to the phyla Bacteroidetes and Proteobacteria [16,17,18]. It is also thought that the severity of antibiotic-induced dysbiosis could be strictly correlated to the effects exerted on some specific groups of colonic bacteria. The post-antibiotic resilience of gut microbiota is indeed thought to be guaranteed, at least partially, by the not-complete disappearance of butyrate-producer genera (e.g., *Faecalibacterium*, *Roseburia*, *Agathobacter*, etc.) and of acetate-producer species (e.g., *Bifidobacterium adolescentis*, *Bifidobacterium catenolatum*, *Bifidobacterium pseudocatenolatum*, etc.), along with a moderate growth of disruptors, such as the putative pathogens belonging to the phylum Proteobacteria (*Escherichia*/*Shigella*, *Klebsiella*, *Enterobacter*, etc.), or “de-novo colonizers” often observed in patients with gut inflammatory diseases, such as *Ruminococcus gnavus*, *Ruminococcus torques*, *Clostridium bolteae*, etc. [19].

Amoxicillin-clavulanic acid (AC), one of the most prescribed antibiotics worldwide, is reported to severely affect the *Bifidobacterium* gut microbiota content both in adults and in children [20,21]. Several studies correlate overweight, atopy, low response to vaccines and autoimmune diseases in children with the drop of bifidobacterial content [9,10,22,23,24,25]. A recent systematic review has shown that the addition of probiotics to antibiotic interventions may partially preserve the alpha diversity and ameliorate the changes in gut microbial composition due to antibiotic interventions [26]. In any case, bacterial probiotics are generally susceptible to most prescribed, orally administered antibiotics, particularly in regards to amoxicillin and AC [27].

Recently, a study analysing hundreds of potential probiotics identified four bifidobacterial strains exhibiting a high level of amoxicillin and AC insensitivity [21]. One of these strains, *Bifidobacterium breve* PRL2020, isolated from a stool sample of one-month breastfed infant born by vaginal birth, showed a MIC (Minimum Inhibitory Concentration) value of 64 µg/mL for amoxicillin and 32 µg/mL for AC. Gut-simulating in vitro experiments revealed that this strain persisted in the presence of a complex microbiota combined with AC. If confirmed, these results could open the possibility (after verifying that horizontal gene transfer of the putative genes determining its antibiotic resistance properties is unlikely to take place) of using this strain in a probiotic product when amoxicillin or AC therapy are prescribed to avoid or limit gut dysbiosis. With this aim, the current study therefore verified the complete strain antibiotic-susceptibility profile, the genomic detection of antibiotic resistance determinants and the presence of mobile elements that could favour the transferability of these resistance features.

## 2. Materials and Methods

### 2.1. Strain and Culturing Media

*B. breve* PRL2020 (LMG S-32458) was subcultured in TOS (transgalactosylated oligosaccharides) propionate agar supplemented with 50 µg/mL (*w*/*v*) of mupirocin (both reagents from Merck Life Science, Darmstadt, Germany), and plates were anaerobically incubated at 37 °C for 72 h. The anaerobic conditions were obtained in the jar using the anaerobic generator (Anaerogen, Thermo Scientific, Milan, Italy) which leads to an O_2_ concentration < 0.1% within 150 min and to a CO_2_ concentration between 7 and 15% within two hours. *B. breve* PRL2020 colonies were grown in MRS broth supplemented with 0.05% L-cysteine hydrochloride (Merck Life Science, Germany) for 48 h at 37 °C under anaerobic conditions.

### 2.2. Antibiotic-Susceptibility Assessment by Microdilution Method

The susceptibility of the tested strain to antibiotics was assessed by MIC determination using the microdilution method. A single aliquot of the liquid culture of *B. breve* PRL2020 was spread onto TOS propionate agar plates. Following incubation in anaerobic conditions as described above, the growth and purity of the strain were checked, and individual colonies were selected and directly resuspended in a tube containing 3 mL of sterile saline solution. Suspensions were prepared to reach a McFarland scale = 1, corresponding to about 3.0 × 10^8^ CFU/mL. This suspension was diluted at the ratio 1:500 in the LSM (lactic acid bacteria susceptibility test medium) broth (MRS from BD, Difco; and ISO SensiTest from Oxoid, Thermofisher, Milan, Italy) supplemented with 0.03% L-cysteine hydrochloride. 100 µL of bacterial suspension were then dispensed within 30 min from the preparation onto precoated SensititreTM EULACBI1 and SensititreTM EULACBI 2 microplates (Thermofisher, Milan, Italy) using a multichannel pipette. The SensititreTM EULACBI1 and SensititreTM EULACBI2 microplates contain the antibiotics listed in the ISO 10932:2010 norm as follows: ampicillin, penicillin, clindamycin, linezolid (range: from 0.03 to 16 μg/mL), vancomycin, ciprofloxacin (range: from 0.25 to 128 μg/mL), neomycin, gentamicin, streptomycin (range: from 0.5 to 256 μg/mL), kanamycin (range: from 2 to 1024 μg/mL), erythromycin, quinupristin-dalfopristin (range: from 0.016 to 8 μg/mL), tetracycline, chloramphenicol, rifampicin, trimethoprim (range: from 0.125 to 64 μg/mL). All antibiotics were purchased from Merck Life Science, Germany. Negative control wells were inoculated with the same sterile medium used for the strain. Sensititre^TM^ microplates were read after 48 ± 3 h of incubation at 37 °C under an anaerobic atmosphere. MIC values were therefore registered. Amoxicillin trihydrate (Merck product number: C10242500EH) and amoxicillin trihydrate-potassium clavulanate (AC) 4:1 (Merck product number: SMB00607) were also considered as additional antibiotics among the tests. The microplate microdilution method was applied. Scalar concentrations of the antibiotic from an original stock solution were manually prepared. Sterile distilled water was used as a diluent. The dilutions were poured onto the microplates together with LSM medium supplemented with cysteine, as described above. A total of ten biological replicates, each with a technical duplicate, were performed. Microplates were incubated for 48 ± 3 h at 37 °C under an anaerobic atmosphere, and MIC values were registered. *B. longum* ATCC 15707 was included within the tests as technical internal control.

### 2.3. Antibiotic-Susceptibility Assessment by Selective Agar Media

Some suspensions of *B. breve* PRL2020 from the highest MIC wells of the ten replicates in microplates, were plated onto TOS-mupirocin propionate agar supplemented with 1–8 μg/mL (*w*/*v*) of clindamycin, 1–16 μg/mL (*w*/*v*) of ampicillin, or 8–128 μg/mL (*w*/*v*) of amoxicillin or AC. Plates were incubated for 48 h at 37 °C under anaerobic conditions.

### 2.4. Species-Specific and Strain-Specific Fingerprints

Colonies isolated from TOS-mupirocin propionate agar plates were lysed by Microlysis PLUS thermal protocol (Microzone, Aurogene, Rome, Italy), and the resulting DNA was used for species-specific and strain-specific PCR amplification. Primers and protocol details for the species-specific PCR were assessed as previously described [28]. *B. breve* ATCC 15700 was included in the species-specific PCR as positive control. Strain-specific profiling was obtained by Rep-PCR amplification. Rep-PCR is a DNA-based molecular technique that allows for the comparison of the genetic profiles of bacterial strains, even if they belong to the same species, to qualitatively assess their polymorphisms. Rep-PCR reactions were conducted using BoxAR1 primer (5′-CTACGGCAAGGCGACCTGACG-3′) as previously described [29]. Thermal cycling and PCR conditions were performed according to the abovementioned paper. PCR amplicons were resolved on agarose gel 2.5% (*w*/*v*) in Tris-acetate-EDTA buffer (TAE). The gels were stained with Midori Green (0.5 μg/mL) (Resnova, Brescia, Italy) and visualized under UV light. The genetic distance between the isolated colonies was visualized, and the strains were clustered based on their overall profiles.

### 2.5. Genomic Analysis of Strain B. breve PRL2020

The genome sequence of the strain *B. breve* PRL2020 was downloaded from NCBI (Genbank Accession Number: JACZEM01.1) and internally archived as MB196. Statistics of the whole genome sequence were obtained from QUAST software v5.0.2.

### 2.6. Identification of Putative Resistance Genes in B. breve PRL2020

To identify known antibiotic resistance (AR) determinants, the annotated genome sequence of *B. breve* PRL2020 (MB196) was employed to query the Comprehensive Antibiotic Resistance Database (CARD version 3.1.4) using Resistance Gene Identifier (RGI) software version 5.2.1 [30,31]. Default parameters were applied for strict and perfect algorithm, specifying the *exclude_nudge* function (https://github.com/arpcard/rgi (accessed on 25 February 2023)—Section: RGI main Usage for Genomes, Genome Assemblies, Metagenomic Contigs, or Proteomes). The loose algorithm was used only for the analysis of AC resistance genes. The similarity among the AR determinants found and those available in public database was studied through ClustalX2 alignment and BLAST searches. In addition, the 30 Kb upstream and downstream regions identified AR determinants, which were analysed by Bionumerics software v.7.6 and BLAST algorithm using IS-finder database [32], to define their composition and to evaluate the horizontal transferability.

## 3. Results

### 3.1. Antibiotic-Resistant Profile of B. breve PRL2020

The test performed with the microdilution method (Table 1) showed that, with regard to gentamicin, the strain PRL2020 demonstrated a MIC value overlapping with that reported by the EFSA (European Food Safety Authority) protocol [33] of 64 µg/mL. Regarding clindamycin, the strain demonstrated a range of values between 0.25 and 16 µg/mL (EFSA cut-off = 1 µg/mL). Regarding ampicillin, the value shown by the strain (8 µg/mL) was also superior to that reported by the EFSA reference (1 µg/mL). All the other MIC values were lower than those reported by the EFSA document or, in cases of no reported EFSA cut-off values, MIC values corresponded with those observed in the technical control (strain ATCC 15707). Regarding amoxicillin and AC, for which reference values are not reported in the EFSA tables and an evaluation with the control strain (ATCC 15707) was not performed, the MIC values were 64 and 32 µg/mL, respectively. The antibiotic susceptibility assessment by selective agar media, performed to obtain a further control of the results obtained with gentamicin, clindamycin, ampicillin, amoxicillin and AC using the microdilution method, confirmed all previous results except for clindamycin, for which the *B. breve* PRL2020 strain demonstrated a MIC of 0.25 µg/mL.

### 3.2. Species-Specific and Strain-Specific Fingerprints

**Figure 1:**
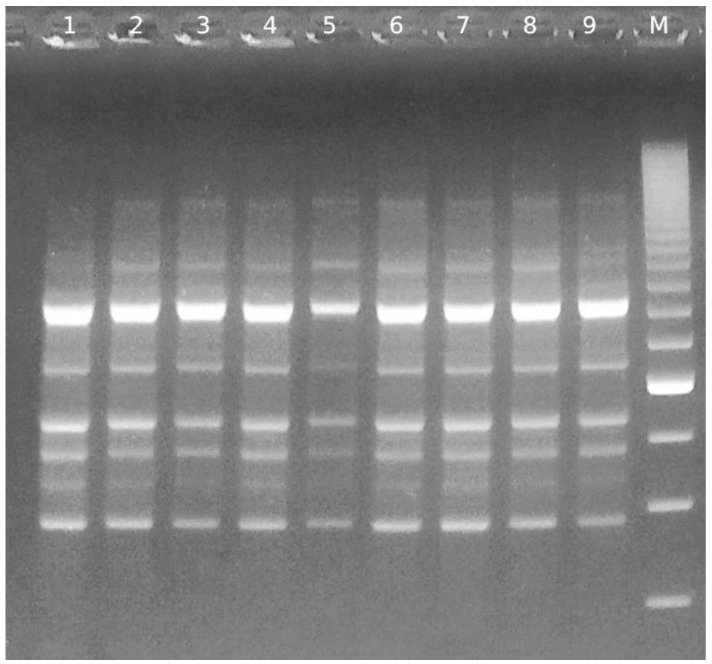
Molecular (BoxAR1) typing results obtained to confirm the singular pattern of different colonies of *B. breve* PRL2020. In lines 1 and 2: PRL2020 grown on TOS-MUP agar plates; in line 3: PRL2020 grown on agar plates with clindamycin at 8 µg/mL; in line 4: PRL2020 grown on agar plates with erythromycin at 0.25 µg/mL; in line 5: PRL2020 grown on agar plates with clindamycin at 4 µg/mL and erythromycin at 0.25 µg/mL; in line 6: PRL2020 grown from titres with erythromycin at 0.25 µg/mL; in line 7: PRL2020 grown from titres with clindamycin at 8 µg/mL; in line 8: PRL2020 grown on TOS-MUP agar plates with amoxicillin at 8 µg/mL; in line 9: PRL2020 grown on TOS-MUP agar plates with amoxicillin at 16 µg/mL. M: marker for 200 bp; TOS: transgalactosylated oligosaccharides; MUP: mupirocin.

Despite the different morphologies displayed by some colonies of the strain *B. breve* PRL2020 in the clindamycin test, all were confirmed to have the same genetic profile of the original culture as shown by the BoxAR1 molecular patterns (Figure 1).

### 3.3. Genome Statistics of B. breve PRL2020

The available genome of the strain *B. breve* PRL2020 (MB196) comprises 2,426,298 bp that were fragmented into six contigs. The number of predicted open reading frames (ORFs) of PRL2020 chromosome consists of 2102 and encompass 54 tRNAs and three rRNA loci. The complete list of its features is described in Table 2.

### 3.4. Identification of Putative Resistance Genes and Analysis of Flanking Regions in B. breve PRL2020

EFSA guidelines recommend that for any bacterial strains to be used as a food probiotic, feed additive or organism production, a genetic investigation must be performed to check for the presence of known antibiotic-resistant (AR) genes [34]. The World Health Organization (WHO) has classified these antibiotics as critically important, highly important and important [35]. By applying both the Perfect and the Strict algorithm with the *exclude_nudge* function (https://github.com/arpcard/rgi (accessed on 25 February 2023)—Section: RGI main Usage for Genomes, Genome Assemblies, Metagenomic Contigs, or Proteomes), the RGI software found four genetic determinants, three of which are potentially involved in the resistance to erythromycin and one to rifampicin (Table 3).

**Figure 2:**
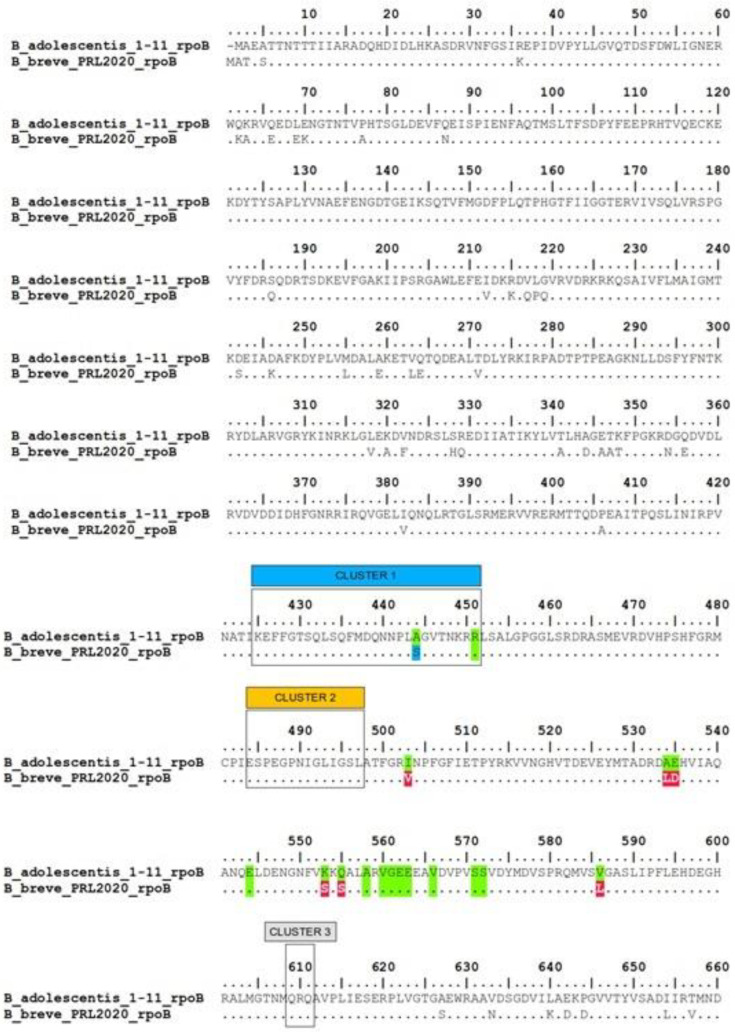
Alignment of the locus IHV18_09970 of *B. breve* PRL2020 with wild-type *B. adolescentis* available in CARD (GenBank Acc. No: WP_041777404.1). The mutation sites conferring rifampicin resistance are highlighted in green, while the mutated residues potentially involved in resistance to rifampicin found in *B. breve* PRL2020 compared to the sequence of *B. adolescentis* are reported in red. Cluster 1, 2 and 3 are highlighted by blue, orange, and grey rectangles, respectively.

Regarding the putative rifampicin-resistant gene, the analysis performed through RGI-CARD on the genome sequence of the strain *B. breve* PRL2020 identified the locus IHV18_09970 (MB196_5.1_683), which codes for a DNA-directed RNA polymerase subunit beta (*rpoB*). Mutations in the rifampicin-binding pocket of *rpoB* inhibit antibiotic activity, leading to the emergence of rifampicin-resistant microorganisms [36]. It has been shown [37] that some *Bifidobacterium* spp. can quickly adapt to different concentrations of rifampicin (from 2 to 100 µg/mL) due to the modification of *rpoB* sequence accumulating mutations in cluster 1 and in the region between cluster 2 and cluster 3 (Figure 2). The alignment between the amino acid sequence of *rpoB* from wild-type *B. adolescentis* available in CARD (GenBank Accession No: WP_041777404.1) and the locus IHV18_09970 showed the presence of one single mutation in the cluster 1 region (depicted in blue in Figure 2) of the strain *B. breve* PRL2020, where alanine (A) at position 443 is substituted by serine (S). The region between cluster 2 and cluster 3 presented six different mutations (reported in red in Figure 2) which were comparable with those found in *Bifidobacterium* spp. treated with rifampicin [37]. In particular: isoleucine (I) at position 502 is mutated in valine (V); alanine (A) at position 533 is mutated in leucine (L); lysine (K) at position 552 is mutated in serine (S); glutamine (Q) at position 554 is mutated in serine (S); valine (V) at position 558 is mutated in leucine (L). Differently from what is observed [37] by exposing *B. adolescentis* to a high concentration of rifampicin (100 µg/mL), the VGEE region (in green in Figure 2) between cluster 2 and cluster 3 (positions 544, 558, 560–563 and 566) and the serine-rich region (positions 570 and 571) are both conserved in the locus IHV18_09970 of *B. breve* PRL2020.

**Figure 3:**
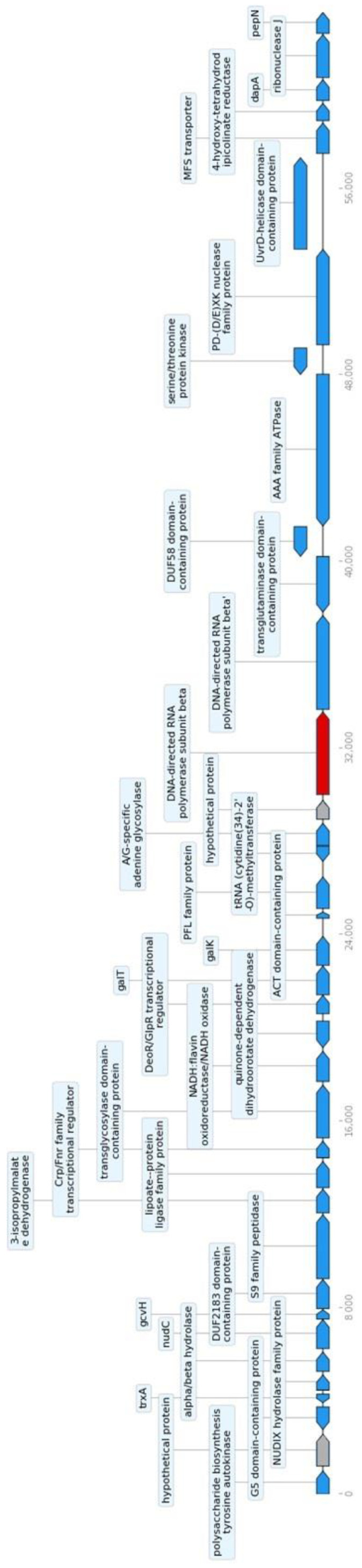
Flanking regions (±30 Kb) of the rpoB gene in *B. breve* PRL2020. The flanking regions (in blue, genes coding for functional proteins) of the locus IHV18_09970 (*rpoB*) (in red), identified by CARD analysis as putative rifampicin resistance determinant, do not reveal the presence of any genes linked to mobile genetic elements within the 60 Kb analysed. In grey, genes coding for hypothetical proteins.

Analysis of the flanking regions for the putative AR determinants identified was carried out to assess the transferability potential of these genes. In fact, the localization of AR determinants on plasmids or near mobile genetic elements, such transposases and/or integrative and conjugative elements (ICEs), could result in the transfer of these genes to other microorganisms. Many bacterial AR have emerged because of genetic changes acquired through mutation or through the uptake of genetic material via horizontal transfer from other bacterial strains [38]. Taking into consideration the average effective size of transposable elements [39], sequences flanking the putative AR genes found in the *B. breve* PRL2020 genome sequence were analysed by retrieving the contigs carrying resistance determinants from the whole genome sequencing data and studying 30 Kb upstream and downstream from the AR gene. The flanking loci with relative positions and gene products are listed in Table 4. The IS-FINDER results for the genetic region located 30 bp downstream and upstream the locus IHV18_09970 (*rpoB*) on contig 5 (BLASTN 2.2.31+, Database: ISfindernt) are listed in Table 5. The performed analysis did not suggest the possible presence of any genes linked to mobile genetic elements within the 60 Kb analysed (Figure 3).

**Figure 4:**
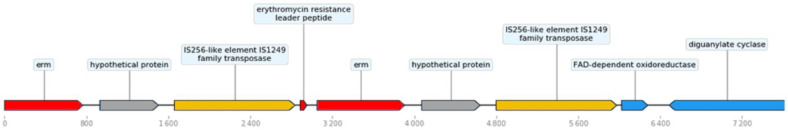
Flanking regions (±30 Kb) of the *erm(X)* gene in *B. breve* PRL2020 (contig 5). Flanking regions of the loci IHV18_06605 and IHV18_06625 [*erm(X)*, in red], which were identified by CARD analysis as putative erythromycin resistance determinants. In blue, genes coding for functional proteins; in yellow, genes coding for transposases; and in grey, genes encoding for hypothetical proteins.

Regarding erythromycin, the analysis performed through RGI-CARD on the genome sequence of the strain *B. breve* PRL2020 identified three copies of the *erm(X)* gene, two loci located on contig 5 and one locus located on contig 4 (Table 3). These loci, IHV18_03440 (MB196_4.1_3), IHV18_06605 (MB196_5.1_1) and IHV18_06625 (MB196_5.1_683) are code for a 23S ribosomal RNA methyltransferase named erm, commonly associated with the *erm(X)* gene, which exhibited a sequence similarity higher than 80% with the reference sequence deposited in CARD known as ARO 300059. The erythromycin-resistant gene *erm(X)* is an antibiotic resistance determinant found in abundance in *Bifidobacterium* sp., where it is often located on the genomic island (BKGI1), which is considered a transferable genetic region [40]. A BLASTn comparison with the nonredundant (nr) database of NCBI revealed a sequence similarity of 99.77% at the nucleotide level for the locus IHV18_03440, with the *erm(X)* gene identified in the strains *Bifidobacterium longum* J3 and *B. longum* SQS7-31, both of which are resistant to erythromycin and clindamycin [41]. The similarity showed by these reference sequences with the loci IHV18_06605 and IHV18_06625 was 98.60%, confirming the integrity of the *erm(X)* determinant for the strain PRL2020. The analysis of the flanking regions for the three copies of the *erm(X)* genes identified in the genome sequence of the strain *B. breve* PRL2020 was carried out as described above for the locus IHV18_09970 and revealed the presence of two flanking CDS, both annotated as an IS256-like element IS1249 family transposase for the locus IHV18_06625 (MB196_5.1_4) (Figure 4 and Table 6).

**Figure 5:**
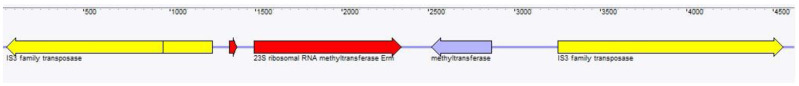
Flanking regions (±30 Kb) of the *erm(X)* gene in *B. breve* PRL2020 (contig 4). Flanking regions of the locus IHV18_03440 [*erm(X)*, in red], which were identified by CARD analysis as putative erythromycin resistance determinants. In blue, genes coding for functional proteins; in yellow, genes coding for transposases; and in grey, genes encoding for hypothetical proteins.

In addition, the IS-FINDER analysis of the region revealed two matching IS1249 hits and two ISCx1 matching the two CDS for hypothetical proteins before the transposase (Table 7). This gene arrangement maps closely to the structure of transposon Tn5432 [41,42,43,44]. The locus IHV18_06605 (MB196_5.1_1) is located just before the previously described Tn5432 transposon, at the very beginning of the sequence of contig 5 (Figure 5).

**Figure 6:**
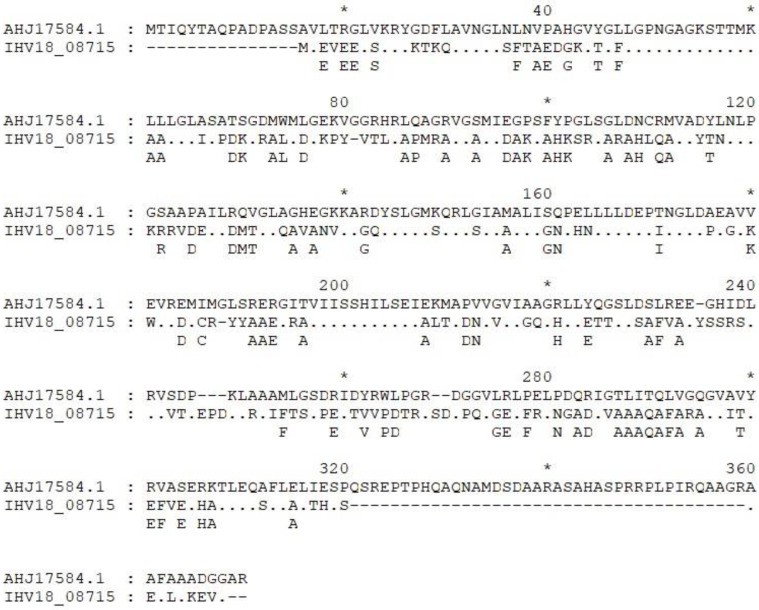
Protein sequence alignment of the locus IHV18_08715 of *B. breve* PRL2020 (MB196), with the bcrA sequence retrieved from the genome sequence of strain *B. breve* JCM 7017.

The evaluation of the flanking region for this locus was limited by its location within the contig. The third locus identified as an *erm(X)* determinant, IHV18_03440 (MB196_4.1_3), is located on contig 4 and is flanked by two transposases belonging to the IS3 family, as reported in Figure 5. Transposons are commonly flanked by terminal inverted repeats; however, these specific regions were not found for either loci IHV18_06625 (MB196_5.1_4) or IHV18_03440 (MB196_4.1_3). Interestingly, a −10-region constituted by the sequence TATAAT was identified upstream the leader peptide of the *erm(X)* gene represented by the locus IHV18_03440 (MB196_4.1_3) but was not identified for the locus IHV18_06625 (MB196_5.1_4). This 6-bp nucleotide sequence together with the −35-region constitute the main components of a typical promoter sequence for the transcription of genetic determinants. Regarding the −35-region, the nucleotide sequence of this part of the promoter for both *erm(X)* genes, loci IHV18_06625 (MB196_5.1_4) and IHV18_03440 (MB196_4.1_3), is not compatible with those previously described for *Bifidobacterium* spp. [45], resulting in a probable lack of promoter function for these AR determinants. According to a previous analysis, the presence of the locus PRL2020_1181 has been described in the strain *B. breve* PRL2020, encoding a predicted ATP-binding cassette (ABC) transporter potentially involved in the observed insensitivity to AC [21]. In addition, two TUGs, ORFs PRL2020_1167 and PRL2020_1282, have also been described to be transcriptionally induced in the strain by the presence of AC, exhibiting a 3.4-fold (*p* value = 0.039; FDR = 0.04) and 2.7-fold (*p* value = 0.049; FDR = 0.044) upregulation, respectively and, in both cases, encoding hypothetical proteins [21]. The authors concluded that these genetic determinants were involved in the resistance of *B. breve* PRL2020 to AC. Interestingly, the comparison with CARD identified the locus PRL2020_1181 as an ABC transporter, conferring resistance to bacitracin in *Bacillus licheniformis* (ARO_3002987—bcrA gene), together with the locus PRL2020_1669 (as described in Table S6 of reference [22]). Due to different annotations of the genome sequences, the locus-tags reported in Table S6 do not match with the ones currently available in the public Genbank file (JACZEM01.1), therefore a direct correlation to determine the localization of the corresponding locus in *B. breve* PRL2020, does not exist. For this reason, the reference protein sequence of the *bcrA* gene (AAA99504.1) from *B. licheniformis* deposited in the CARD database was used to retrieve the homologous sequence from the complete genome of *B. breve* JCM 7017 (AHJ17584) using BLASTp. Subsequently, a BLASTp search against the genome sequence of the strain *B. breve* PRL2020 was carried out using the bcrA protein sequence retrieved from *B. breve* JCM 7017 (AHJ17584). Seven corresponding sequences were found in *B. breve* PRL2020, with their identity ranging from 39.05 to 27.36% (Table 8). The locus IHV18_08715 exhibited the highest identity with the protein sequence of the *bcrA* gene in *B. breve* JCM 7017 (Figure 6). In addition, the seven loci identified through BLASTp analysis and reported in Table 7 were analysed through the CARD database, retrieving bcrA ABC antibiotic efflux pump (ARO:3002987) as the best hit (Table 9). However, the identity at the amino acid level was much lower than 80%, which is considered the threshold for the assignment of a genetic determinant to a putative antibiotic resistance function based on the EFSA guidelines [46]. To verify the presence of additional genetic determinants described as possible AC resistance genes [21], the annotated genome sequence of the strain *B. breve* PRL2020 was analysed through RGI software v.5.2.1 by applying the lose algorithm to query CARD. This analysis retrieved different hits potentially linked to the genes *macB*, *mepA*, *novA*, *PmrF*, *tet(38)*, *vanSO*, and *AdeN*. However, the identity at the amino acid level was much lower than 80% (similar to that observed for the *brcA* genetic determinant, see Supplementary Table S1). These findings clearly indicate the impossibility of determining the localization of the putative genes assumed to be responsible for the AC-resistant features of strain *B. breve* PRL2020. Last, no sequences have been found that can explain the low sensitivity to ampicillin of the strain *B. breve* PRL2020 in the antibiotic susceptibility assessments.

## 4. Discussion

The strain *B. breve* PRL2020 has been isolated from a stool sample of one-month-old infant during a large bifidobacterial survey study directed to investigate the autochthonous members of the bifidobacterial population residing in the human intestine of healthy subjects. *B. breve* PRL2020 was precisely identified by the sequencing of the 16S rRNA gene. This result was corroborated using several housekeeping genes, such as *clpC*, *dnaB*, *dnaG*, *dnaJ1*, *purF*, *rpoC*, and *xfp*, which represents a currently recognized multilocus approach for appropriated bifidobacterial identification at the species level as well as an understanding of bifidobacterial evolution [47]. Considering that almost 99% of bifidobacterial species are considered sensitive, the peculiarity of this strain is reported to be its amoxicillin and AC insensitivity [21]. The authors identified some gene candidates thought to be responsible for high resistance to these two antibiotics [21]. After verifying the complete profile of its possible antibiotic-resistant properties, the authors also decided to analyse if horizontal gene transfer of these assumed genes was unlikely to take place. This aspect is in fact fundamental to exclude since genomic antibiotic resistance can transfer from the probiotic, administered during the antibiotic therapy, to the potential pathogen already present in the context of the microbiota of the host treated with the antibiotic, even if only putative. The transfer of genetic material capable of inducing resistance to one or more antibiotics is indeed activated first when the antibiotic pressure grows [4,5].

Our tests, performed first with the microdilution method and then with the antibiotic susceptibility assessment using selective agar media (while also checking the BoxAR1 molecular patterns of the colonies), demonstrated that (i) the strain *B. breve* PRL2020 is sensitive to gentamicin with a MIC value corresponding with that reported by the EFSA protocol; (ii) the strain is resistant to ampicillin with a value of 8 µg/mL, 8 times superior to that reported by EFSA as reference; (iii) the strain is very sensitive to clindamycin when tested in selective agar media, in spite of the fact that the microdilution method showed that few colonies are less sensitive; (iv) the molecular patterns of colonies that show a different phenotype versus clindamycin have the same genetic profile of the original culture; (v) the strain is very sensitive to all the other antibiotics tested and reported by the EFSA guidelines; and (vi) the strain is insensitive to amoxicillin, AC, and antibiotics not evaluated by the EFSA guidelines with MIC values of 64 and 32 µg/mL, respectively.

Using deep genomic analysis, we have tried to identify all the possible resistance genes in addition to analysing the flanking regions of the putative AR genes detected. Our results have demonstrated that: (i) although it resulted to be very sensitive to rifampicin with a MIC value of <0.12 µg/mL, the strain PRL2020 showed the presence of a *rpoB* mutant gene (homology > 92%, Table 3) which is potentially involved in the resistance to rifampicin; (ii) the total absence of any genes linked to mobile genetic elements within the 30 Kb upstream and downstream the *rpoB* mutant gene; (iii) although it resulted to be phenotypically susceptible to erythromycin with a MIC value of 0.25 µg/mL, three *erm(X)* genes (homology >80%, Table 3) conferring resistance to erythromycin have been found in the genome sequence of the strain; (iv) the presence in the 30 Kb upstream and downstream the *erm(X)* gene of mobile elements identified as transposase of the IS1249 and of the IS3 families (Figure 4 and Figure 5); (v) the absence of genes that could explain the PRL2020 features of low sensitivity to gentamicin and of insensitivity to ampicillin, amoxicillin and AC, which, then, should be considered intrinsic and not genetically transferable.

For ampicillin, an antibiotic to which *Bifidobacterium* strains are usually susceptible [48], a recent study [49] demonstrated that an increase in the production of exopolysaccharides in bifidobacterial cells causes an enhancement in the tolerance toward various beta-lactam antibiotics. Therefore, the peculiar composition of the external membrane of the cell could explain phenotypic resistance, for which there is no direct link with genetic determinants.

Additionally, considering the issue of the *erm(X)* genes and their mobile elements, these results suggest a possible probiotic use for the strain PRL2020. In fact, the performed tests (see Table 1) have clearly demonstrated that the strain is susceptible to erythromycin at four times less than the EFSA cut-off [33]. We have also taken into consideration that the presence of the *erm(X)* gene is widespread among bifidobacterial strains, especially considering *B. breve* and *B. longum*. Analysing the probiotic strains whose genome is available in a public database shows that those carrying the same gene responsible for the erythromycin resistance are 20 for *B. breve* and 61 for *B. longum* (Supplementary Table S2). Since in bifidobacterial species, the rate of erythromycin resistance is directly proportional to the fold-change expression of the *erm(X)* gene [45], it is likely that the expression in *B. breve* PRL2020 is poor. In fact, based on the analysis of the nucleotide regions located upstream the leader peptide of the *erm(X)* genes, the absence of −35 and −10-promoter elements might affect the proper expression of these AR determinants, resulting in a susceptible erythromycin phenotype for the strain *B. breve* PRL2020 despite the presence of *erm(X)* genes in its genome sequence. Similarly, Wang et al. correlate the moderate macrolide resistance displayed by the strain *B. longum* Y2 to a nucleotide transition in the −35-promoter region, resulting in a low level of expression of the *erm(X)* gene [45].

Finally, as observed in filter mating experiments, in bifidobacterial species, the transfer of the *erm(X)* gene occurs only when the gene is simultaneously flanked by IS1249 and IS3 elements. Otherwise, if the gene is individually flanked by IS1249 or IS3, the transfer does not occur. This indicates the need for a synergic effect of IS1249 and IS3 elements in the transfer of *erm(X)* in *Bifidobacterium* species [45]. We have clearly observed that for the two copies of *erm(X)* present in contig 5, only flanking elements of the IS1249 family are present. Similarly, for the single copy in contig 4, only flanking elements of IS3 family are present. However, a genomic island of 55 Kb named BKGI1 it has been described that mediates the transfer of *erm(X)* gene inserted in a Tn5432 transposon from *Bifidobacterium catenulatum* subsp. *kashiwanohense* DSM 21854 to *Bifidobacterium longum* subsp. *suis* DSM 20211, even though the Tn5432 carrying the *erm(X)* gene contains two t IS1249 transposase [40]. The BKGI1 excises from the chromosome, forms a circular intermediate, transfers by conjugation and, once in the recipient cell, can completely integrate with the chromosome or the transposon Tn5432 can excise from the genomic island and integrate itself in the chromosome of the recipient. In the latter case, it seems that the transposon Tn5432 lacks the capability to transfer to other microorganisms once excised from the genomic island and integrated in the chromosome [40]. Analysing the regions flanking the *erm(X)* genes for the strain *B. breve* PRL2020, it does not provide any evidence of the presence of the complete sequence of this genomic island of 55 Kb, supporting the non-transferability of these genetic determinants. Based on the results described in this study, the observed features of antibiotic insensitivity demonstrated by the strain *B. breve* PRL2020 (ampicillin, amoxicillin, and AC) appear not to be genetically supported and therefore should not be considered horizontally transferable. The use of this strain as a probiotic, when concomitantly administered with antibiotics like ampicillin, amoxicillin, and AC, could be considered an innovative therapeutical approach aimed to reduce gut dysbiosis, at least partially.

Other examples of this approach of using a specific AR strain to reduce specific antibiotic-induced dysbiosis have been demonstrated. For instance, the strain *B. longum* W11 (LMG P-21586) was not inhibited by rifaximin until the concentration of 512 mg/mL. The genomic analysis showed a mutation (*rpoB*) into the chromosomal DNA. No transposable elements were found and the genetic locus was not flanked by close mobile genetic elements [50,51]. In medical practice, the use of rifaximin along with probiotics is quite common in patients with a diagnosis of symptomatic uncomplicated diverticular disease (SUDD), with the latter being administered at the end of the rifaximin cycle. The opportunity of having a probiotic strain like *B. longum* W11 described as being resistant to rifaximin suggested to clinicians its use in subjects with SUDD, administering it concomitantly with rifaximin. Indeed, patients treated with rifaximin concomitantly receiving strain W11 demonstrated to be more gut-dysbiosis-resilient and showed better clinical outcomes than control subjects [52].

As for *B. breve* PRL2020, clinical data have not yet demonstrated the advantages of this type of approach in reducing possible specific antibiotic-mediated gut dysbiosis. In fact, only experiments on simulating gut microbiota have revealed that the PRL2020 strain can survive in the presence of a complex microbiota combined with a specific AC antibiotic [21]. Furthermore, recent in vivo studies on rodent models have confirmed the ability of AC-resistant bifidobacterial strains (a less AC-resistant than the strain *B. breve* PRL2020) to bolster gut microbiota resilience, increase biodiversity, preserve gut microbiota eubiosis and prevent bifidobacterial strains from disappearing, revealing strain-specific and a strain-non-specific impacts (possibly due to the covariance phenomena on the microbiota composition of bifidobacterial taxa) [53].

## 5. Conclusions

The results of our tests have demonstrated that the strain PRL2020 is sensitive to all the antibiotics tested except amoxicillin, AC, and ampicillin. Deep genome analysis has previously shown the absence of any genes explaining these features and must therefore be considered intrinsic and non-transferable. Deep genome analysis has also shown the presence of genetic sequences (*rpoB* and *erm(X)*) putatively responsible for rifampicin and erythromycin resistance. Both these features are not phenotypically expressed as being the strain within the EFSA limits. Analysis of the flanking regions of these two genes revealed possible mobile elements upstream and downstream only in the case of the *erm(X)* gene, but the features of the Insertion Sequences (IS) are described as not causing horizontal transfer. The results provided by our analysis of the strain *B. breve* PRL2020 demonstrate that its AR profile is compatible when taken with specific antibiotics with the aim of reducing the risk of antibiotic-caused dysbiosis.

## Figures and Tables

**Table 1:** MIC values (expressed as µg/mL) for *B. breve* PRL2020 and *B. longum* ATCC 15707 (technical control).

Antibiotic	PRL2020	ATCC 15707	EFSA Cut-Off
Gentamicin	64	32	64
Kanamycin	512	512	
Streptomycin	64	64	128
Neomycin	64	64	
Tetracycline	1	2	8
Erythromycin	0.25	0.25	1
Clindamycin	0.25–16	0.03	1
Chloramphenicol	2	4	4
Ampicillin	8	1	2
Penicillin	4	0.2	
Vancomycin	<0.25	0.5	2
Quinupristin-Dalfopristin	0.5	0.25	
Linezolid	1	1	
Trimethoprim	>64	32	
Ciprofloxacin	16	16	
Rifampicin	<0.12	0.12	
Amoxicillin/Clavulanic acid	32	not done	
Amoxicillin	64	not done	

EFSA: European Food Safety Authority.

**Table 2:** Statistics for the whole genome sequence of the strain *B. breve* PRL2020 (MB196).

Features	*B. breve* PRL2020 (MB196)
Number of scaffolds	6
Genome size (bp)	2,426,298
Maximum contig (bp)	986,561
Minimum contig (bp)	1,944,282
Average scaffold (bp)	5020
GC content (%)	59.10
N50	729,119
N75	575,136
L50	2
L75	3
Number of open reading frames (ORFs)	2102
Number of tRNA	54
Number of rRNA loci	3

N50 and N75: the sequence length of the shortest contig at 50% and 75% of the total assembly length, respectively. L50 and L75: the smallest number of contigs whose length sum makes up 50% and 75% of the total genome size, respectively.

**Table 3:** Antibiotic resistance determinants identified in *B. breve* PRL2020 performed through the comparison against CARD, in which the reference sequences are organized by the Antibiotic Resistance Ontology (ARO), and they are classified in AMR (antimicrobial resistance) genes families.

Locus Tag	Annotation	Algorithm	Best Hit CARD	ARO	AMR Gene Family	Identity (%)	Coverage (%)
IHV18_03440	23S ribosomal RNA methyltransferase erm	Strict	*Erm(X)*	3000596	Erm 23S ribosomal RNA methyltransferase	86.97	100.00
IHV18_06605	23S ribosomal RNA methyltransferase erm	Strict	*Erm(X)*	3000596	Erm 23S ribosomal RNA methyltransferase	87.84	89.79
IHV18_06625	23S ribosomal RNA methyltransferase erm	Strict	*Erm(X)*	3000596	Erm 23S ribosomal RNA methyltransferase	88.03	100.00
IHV18_09970	DNA-directed RNA polymerase subunit beta	Strict	*B. adolescentis rpoB* mutants conferring resistance to rifampicin	3004480	rifamycin-resistant beta-subunit of RNA polymerase (*rpoB*)	92.48	100.00

**Table 4:** Loci present in the flanking regions of locus IHV18_09970 (reported in bold) of *B. breve* PRL2020.

Locus Tag	Gene	Start	End	Strand	Product
IHV18_09850		<0	1005	1	polysaccharide biosynthesis tyrosine autokinase
IHV18_09855		1202	2579	1	hypothetical protein
IHV18_09860		2766	3750	−1	G5 domain-containing protein
IHV18_09865	*trxA*	3922	4294	−1	thioredoxin
IHV18_09870		4461	5124	1	NUDIX hydrolase family protein
IHV18_09875		5259	6105	1	alpha/beta hydrolase
IHV18_09880	*nudC*	6238	7495	1	NAD(+) diphosphatase
IHV18_09885	*gcvH*	7510	7912	1	glycine cleavage system protein GcvH
IHV18_09890		7958	9215	1	DUF2183 domain-containing protein
IHV18_09895		9237	11,994	1	S9 family peptidase
IHV18_09900		12,060	13,092	1	3-isopropylmalate dehydrogenase
IHV18_09905		13,142	14,276	1	lipoate–protein ligase family protein
IHV18_09910		14,428	15,148	1	Crp/Fnr family transcriptional regulator
IHV18_09915		15,276	17,553	1	transglycosylase domain-containing protein
IHV18_09920		17,697	19,002	1	NADH:flavin oxidoreductase/NADH oxidase
IHV18_09925		19,146	20,298	−1	quinone-dependent dihydroorotate dehydrogenase
IHV18_09930		20,602	21,406	1	DeoR/GlpR transcriptional regulator
IHV18_09935	*galT*	21,411	22,662	1	galactose-1-phosphate uridylyltransferase
IHV18_09940	*galK*	22,679	23,930	1	galactokinase
IHV18_09945		24,698	24,971	1	ACT domain-containing protein
IHV18_09950		25,115	26,480	1	PFL family protein
IHV18_09955		27,138	27,801	−1	tRNA (cytidine(34)-2′-O)-methyltransferase
IHV18_09960		27,821	28,784	1	A/G-specific adenine glycosylase
IHV18_09965		28,947	29,766	1	hypothetical protein
**IHV18_09970**		**30,000**	**33,564**	**1**	**DNA-directed RNA polymerase subunit beta**
IHV18_09975		33,654	37,692	1	DNA-directed RNA polymerase subunit beta’
IHV18_09980		37,830	40,221	−1	transglutaminase domain-containing protein
IHV18_09985		40,217	41,507	−1	DUF58 domain-containing protein
IHV18_09990		41,520	48,045	−1	AAA family ATPase
IHV18_09995		48,016	49,165	−1	serine/threonine protein kinase
IHV18_10000		49,301	53,399	1	PD-(D/E)XK nuclease family protein
IHV18_10005		53,395	57,331	1	UvrD-helicase domain-containing protein
IHV18_10010		57,502	58,822	1	MFS transporter
IHV18_10015		58,909	59,665	1	4-hydroxy-tetrahydrodipicolinate reductase
IHV18_10020	*dapA*	59,770	60,676	1	4-hydroxy-tetrahydrodipicolinate synthase
IHV18_10025		60,755	62,612	1	ribonuclease J
IHV18_10030	*pepN*	62,653	>63,564	1	aminopeptidase N

**Table 5:** IS-FINDER results for the genetic region located 30 Kbp downstream and upstream the locus IHV18_09970 (*rpoB*) on contig 5.

Subj.id	% id	Ali.len.	Mism.	Gaps	Q.start	Q.end	S.start	S.end	E-Value	Bit Score
ISPye9	100.00	20	0	0	23,639	23,658	1006	987	0.44	40.1
ISFsp17	100.00	20	0	0	22,409	22,428	1224	1243	0.44	40.1
ISAtu2	100.00	20	0	0	6124	6143	1328	1309	0.44	40.1
ISAtu2	100.00	20	0	0	5148	5167	1328	1309	0.44	40.1
TnXo19	100.00	19	0	0	48,445	48,463	8947	8965	1.7	38.2
TnXo19	100.00	18	0	0	50,737	50,754	1226	1209	6.9	36.2
ISFK1	100.00	19	0	0	44,600	44,618	2326	2308	1.7	38.2
ISSba13	100.00	19	0	0	45,366	45,384	1244	1226	1.7	38.2
ISSpu6	100.00	19	0	0	45,366	45,384	1244	1226	1.7	38.2
ISLxc1	100.00	19	0	0	38,963	38,981	1990	1972	1.7	38.2
ISYen2A	100.00	19	0	0	24,512	24,530	1600	1618	1.7	38.2
ISYen2B	100.00	19	0	0	24,512	24,530	1594	1612	1.7	38.2
ISPna2	95.65	23	1	0	33,302	33,324	836	858	1.7	38.2
ISRso19	100.00	19	0	0	19,826	19,844	1401	1383	1.7	38.2
ISKpn64	100.00	18	0	0	19,036	19,053	2039	2056	6.9	36.2
ISArsp9	100.00	18	0	0	2907	2924	1643	1660	6.9	36.2
ISAcp4	100.00	18	0	0	58,347	58,364	705	688	6.9	36.2
ISPye36	100.00	18	0	0	37,215	37,232	1000	1017	6.9	36.2
ISJsp3	100.00	18	0	0	26,055	26,072	721	738	6.9	36.2
ISMmo1	100.00	18	0	0	42,773	42,790	1100	1083	6.9	36.2
ISHla15	100.00	18	0	0	35,492	35,509	1402	1385	6.9	36.2
ISNpe19	100.00	18	0	0	60,255	60,272	366	383	6.9	36.2
ISBibr1	95.45	22	1	0	16,247	16,268	1429	1408	6.9	36.2
ISPa43	95.45	22	1	0	33,419	33,440	11,925	11,946	6.9	36.2
ISMch9	100.00	18	0	0	34,809	34,826	542	559	6.9	36.2
ISGeob1	100.00	18	0	0	42,067	42,084	468	451	6.9	36.2
ISRjo3	100.00	18	0	0	23,575	23,592	1296	1313	6.9	36.2
ISAzo2	100.00	18	0	0	23,776	23,793	821	804	6.9	36.2
ISAzo1	100.00	18	0	0	23,770	23,787	876	893	6.9	36.2
ISCc5	100.00	18	0	0	19,508	19,525	961	944	6.9	36.2
ISStma11	100.00	18	0	0	1906	1923	3426	3409	6.9	36.2
ISTha1	95.45	22	1	0	6819	6840	1248	1227	6.9	36.2
ISRm32	100.00	18	0	0	55,038	55,055	737	754	6.9	36.2
ISGdi10	100.00	18	0	0	35,915	35,932	554	571	6.9	36.2
ISPst7	100.00	18	0	0	34,294	34,311	1117	1100	6.9	36.2
ISGur11	100.00	18	0	0	63,108	63,125	933	950	6.9	36.2
ISAzvi12	92.31	26	2	0	32,448	32,473	487	512	6.9	36.2
ISMav4	95.45	22	1	0	53,178	53,199	223	202	6.9	36.2
ISRhosp3	100.00	18	0	0	1276	1293	982	999	6.9	36.2
ISBvi1	100.00	18	0	0	15,078	15,095	465	482	6.9	36.2
ISNeu3	100.00	18	0	0	47,985	48,002	632	649	6.9	36.2
ISSfl8	100.00	18	0	0	11,367	11,384	974	991	6.9	36.2
ISRtr1	100.00	18	0	0	51,045	51,062	139	156	6.9	36.2
ISPa42	100.00	18	0	0	34,234	34,251	6551	6568	6.9	36.2
ISCre1	100.00	18	0	0	31,600	31,617	522	539	6.9	36.2
IS406	100.00	18	0	0	5152	5169	62	79	6.9	36.2
IS406	100.00	18	0	0	6128	6145	62	79	6.9	36.2
IS1137	100.00	18	0	0	53,291	53,308	425	408	6.9	36.2

Subj.id: reference accession number in IS-FINDER. % id: percentage of identities. Ali.len: alignment length. Mism: number of mismatches. Q.start: start of alignment in query. Q.end: end of alignment in query. S. start: start of alignment in subject. S.end: end of alignment in subject.

**Table 6:** Loci present in the flanking regions of the loci IHV18_06605 and IHV18_06625 (reported in bold).

Locus_Tag	Gene	Start	End	Strand	Product	Notes
**IHV18_06605**	**erm**	**<0**	**768**	**1**	**23S ribosomal RNA methyltransferase erm**	
IHV18_06610		932	1505	1	hypothetical protein	
IHV18_06615		1659	2838	1	IS256-like element IS1249 family transposase	Tn5432
IHV18_06620		2888	2954	1	erythromycin resistance leader peptide	
**IHV18_06625**	**erm**	**3053**	**3908**	**1**	**23S ribosomal RNA methyltransferase erm**	
IHV18_06630		4072	4645	1	hypothetical protein	
IHV18_06635		4799	5978	1	IS256-like element IS1249 family transposase	
IHV18_06640		6024	6282	1	FAD-dependent oxidoreductase	
IHV18_06645		6487	7615	1	diguanylate cyclase	

**Table 7:** IS-FINDER results for contig 5 harbouring the loci IHV18_06605 and IHV18_06625.

Subj.id	% id	Ali.len.	Mism.	Gaps	Q.start	Q.end	S.start	S.end	E-Value	Bit Score
IS1249	100.00	1385	0	0	1499	2883	1	1385	0.0	2746
IS1249	100.00	1385	0	0	4639	6023	1	1385	0.0	2746
ISCx1	100.00	536	0	0	807	1342	1	536	0.0	1063
ISCx1	100.00	536	0	0	3947	4482	1	536	0.0	1063
ISBad2	89.72	1479	143	2	773,043	774,521	1470	1	0.0	1725
ISBlo8	98.30	1408	6	1	975,494	976,883	1408	1	0.0	2627
ISBlo8	98.22	1408	7	1	79,047	80,436	1	1408	0.0	2619
ISBlo8	98.22	1408	7	1	47,706	49,095	1408	1	0.0	2619
ISBlo7	94.16	1267	73	1	266,411	267,676	1267	1	0.0	1917
ISBlo7	91.01	979	88	0	78,043	79,021	174	1152	0.0	1243
ISCre1	81.72	930	168	2	1955	2883	457	1385	2 × 10^−130^	480
ISCre1	81.72	930	168	2	5095	6023	457	1385	2 × 10^−130^	480
IS3507	87.89	925	110	1	1950	2872	453	1377	0.0	940
IS3507	87.89	925	110	1	5090	6012	453	1377	0.0	940
ISCge1	88.65	916	104	0	1957	2872	460	1375	0.0	991
ISCge1	88.65	916	104	0	5097	6012	460	1375	0.0	991
ISBad1	97.45	707	16	1	759,078	759,784	2524	1820	0.0	1253
ISBlo9	84.03	595	95	0	759,784	760,378	1	595	3 × 10^−115^	426
ISBlo3	84.12	466	74	0	773,099	773,564	1423	958	2 × 10^−87^	337
ISBlo2	85.39	445	65	0	63,104	63,548	1908	2352	2 × 10^−96^	367
ISBlo2	85.17	445	66	0	756,446	756,890	1908	2352	6 × 10^−94^	359

Subj.id: reference accession number in IS-FINDER. % id: percentage of identities. Ali.len: alignment length. Mism: number of mismatches. Q.start: start of alignment in query. Q.end: end of alignment in query. S. start: start of alignment in subject. S.end: end of alignment in subject.

**Table 8:** BLASTp results using the *bcrA* sequence from *B. breve* JCM 7017 (AHJ17584) as queried against the genome sequence of the strain *B. breve* PRL2020.

Locus Tag	GenBank Accession Number	Contig	Start	Stop	Strand	Bitscore	Identity (%)
IHV18_08715	MBK5036329.1	JACZEM010000005.1_425	548,292	549,230	+	140.6	39.05
IHV18_05290	MBK5035713.1	JACZEM010000004.1_370	448,256	448,912	−	119.8	33.17
IHV18_00625	MBK5034859.1	JACZEM010000001.1_126	143,207	144,079	−	86.7	31.71
IHV18_09555	MBK5036482.1	JACZEM010000005.1_591	710,345	711,754	−	107.5	30.74
IHV18_05265	MBK5035708.1	JACZEM010000004.1_366	443,545	444,417	−	89.4	29.36
IHV18_05000	MBK5035662.1	JACZEM010000004.1_318	389,296	390,000	−	93.2	27.98
IHV18_00430	MBK5034821.1	JACZEM010000001.1_88	97,807	98,727	+	115.2	27.36

**Table 9:** BLASTp results for the loci of *B. breve* PRL2020 reported in Table 7 against CARD.

Locus Tag	Bitscore	ARO Tag	Name	E-Value	Identity (%)	Species
IHV18_08715	148	ARO:3002987	bcrA	2 × 10^−42^	39	*Bacillus licheniformis*
IHV18_05290	118	ARO:3002987	bcrA	2 × 10^−32^	33	*Bacillus licheniformis*
IHV18_00625	89	ARO:3002987	bcrA	2 × 10^−21^	31	*Bacillus licheniformis*
IHV18_09555	107	ARO:3002987	bcrA	8 × 10^−27^	30	*Bacillus licheniformis*
IHV18_05265	90	ARO:3002987	bcrA	5 × 10^−22^	30	*Bacillus licheniformis*
IHV18_05000	92	ARO:3002987	bcrA	8 × 10^−23^	28	*Bacillus licheniformis*
IHV18_00430	109	ARO:3002987	bcrA	3 × 10^−28^	27	*Bacillus licheniformis*

ARO: Antibiotic Resistance Ontology.

## Data Availability

Data related to this manuscript can be made available from the corresponding author upon reasonable request.

## Supplementary Materials

The following supporting information can be downloaded at: https://www.mdpi.com/article/10.3390/microorganisms11071649/s1. Table S1: List of genetic determinants identified through the analysis carried out with RGI software against CARD database using the Loose algorithm for the strain *B. breve* PRL2020; Table S2: Description of the genome sequences available in public database for *B. breve*, which harbour *erm(X)* and *erm(D)* genes.
