# Supplementary material for: Bifidobacterium breve PRL2020: Antibiotic-Resistant Profile and Genomic Detection of Antibiotic Resistance Determinants
Source: Microorganisms. 2023 Jun 24;11(7):1649. doi: 10.3390/microorganisms11071649 (PMC10383950; doi:10.3390/microorganisms11071649)
Supplement: Supplementary file 1 [file microorganisms-11-01649-s001.zip › Supplementary Table S1.pdf]

Supplementary Table S1. List of genetic determinants identified through the analysis carried out with RGI software against CARD database using the Loose algorithm for the strain *B. breve* PRL2020.

AR gene	Contig	Start	Stop	Orientation	Cut_Off	PassBS	BestHBS	BHARO	BestIdent
macB	JACZEM010000001.1_273	311445	312356	+	Loose	1280	85.9	macB	46.88
	JACZEM010000001.1_199	231412	232338	+	Loose	1280	188.7	macB	44.8
	JACZEM010000001.1_531	565157	565837	+	Loose	1280	151.4	macB	42.44
	JACZEM010000005.1_757	916950	917555	-	Loose	1280	104.0	macB	41.01
	JACZEM010000004.1_608	712947	713864	-	Loose	1280	145.6	macB	40.2
	JACZEM010000004.1_203	242223	243086	-	Loose	1280	150.2	macB	39.56
	JACZEM010000005.1_747	906664	907476	+	Loose	1280	181.4	macB	39.37
	JACZEM010000005.1_387	500254	501051	+	Loose	1280	175.3	macB	38.91
	JACZEM010000005.1_700	851689	852885	+	Loose	1280	156.0	macB	38.84
	JACZEM010000001.1_171	198386	199588	+	Loose	1280	134.8	macB	37.31
	JACZEM010000004.1_422	502187	502822	+	Loose	1280	121.3	macB	36.97
	JACZEM010000005.1_721	877063	877848	+	Loose	1280	110.5	macB	36.44
	JACZEM010000005.1_555	671704	672414	-	Loose	1280	116.7	macB	36.24
	JACZEM010000005.1_107	134046	134747	-	Loose	1280	157.9	macB	35.59
	JACZEM010000005.1_707	859144	859974	+	Loose	1280	131.7	macB	35.42
	JACZEM010000005.1_720	876279	877070	+	Loose	1280	102.4	macB	34.55
	JACZEM010000005.1_802	967184	969721	+	Loose	1280	46.6	macB	34.07
	JACZEM010000005.1_749	908795	910324	+	Loose	1280	74.7	macB	33.14
	JACZEM010000005.1_91	108793	110931	-	Loose	1280	101.7	macB	32.66
	JACZEM010000001.1_200	232370	235108	+	Loose	1280	50.8	macB	31.62
	JACZEM010000004.1_186	222877	225228	+	Loose	1280	99.8	macB	31.22
	JACZEM010000001.1_272	310226	311443	+	Loose	1280	39.3	macB	30.65
macB	JACZEM010000001.1_532	565834	567003	+	Loose	1280	39.3	macB	30.34
	JACZEM010000001.1_396	441589	442290	-	Loose	1280	64.7	macB	30.14
	JACZEM010000004.1_374	451378	452106	-	Loose	1280	89.4	macB	27.32
	JACZEM010000005.1_71	82130	83314	+	Loose	1280	50.1	macB	26.86
mepA	JACZEM010000001.1_215	248076	249044	+	Loose	850	97.1	mepA	25.41
novA	JACZEM010000005.1_76	89537	91342	-	Loose	1190	339.7	novA	35.15
	JACZEM010000001.1_5	5114	5824	-	Loose	1190	80.5	novA	32.37
	JACZEM010000001.1_315	351387	352166	+	Loose	1190	69.7	novA	30.22
	JACZEM010000005.1_600	722467	726528	+	Loose	1190	151.8	novA	27.24

**PmrF**	JACZEM010000005.1_59	64991	65959	-	Loose	550	169.1	PmrF	32.48
	JACZEM010000005.1_621	750761	751108	-	Loose	550	41.6	PmrF	25.81
**Tet(38)**	JACZEM010000004.1_260	310067	311467	-	Loose	850	186.8	tet(38)	27.68
**vanSO**	JACZEM010000004.1_379	455194	456495	+	Loose	650	106.3	vanSO	
**AdeN**	JACZEM010000005.1_400	519648	520496	-	Loose	420	49.7	adeN	43.33
	JACZEM010000005.1_332	432055	432654	-	Loose	420	47.8	adeN	32.31
