# Supplementary material for: Bifidobacterium breve PRL2020: Antibiotic-Resistant Profile and Genomic Detection of Antibiotic Resistance Determinants
Source: Microorganisms. 2023 Jun 24;11(7):1649. doi: 10.3390/microorganisms11071649 (PMC10383950; doi:10.3390/microorganisms11071649)
Supplement: Supplementary file 1 [file microorganisms-11-01649-s001.zip › Supplementary Table S2.pdf]

**Supplementary Table S2. Description of the genome sequences available in public database for *B. breve*, which harbour *erm*(X) and *erm*(D) genes.**

| Species | Strain | Genome Acc. No. | Authors and link | Affiliation |
|---|---|---|---|---|
| *Bifidobacterium breve* | MCC 1605 | NZ_AWFV01000006.1 | Odamaki,T., Horigome,A., Sugahara,H., Hashikura,N., Minami,J.,Xiao,J.Z. and Abe,F.<br>https://www.ncbi.nlm.nih.gov/pmc/articles/PMC4506816/ | Morinaga Milk Industry Co. |
| *Bifidobacterium breve* | BR-06 | NZ_BCXL01000075.1 | Matsuki,T., Yahagi,K., Mori,H., Matsumoto,H., Hara,T., Tajima,S., Ogawa,E., Kodama,H., Yamamoto,K., Yamada,T., Matsumoto,S. and Kurokawa,K.<br>https://pubmed.ncbi.nlm.nih.gov/27340092/ | Yakult Central Institute |
| *Bifidobacterium breve* | BR-10 | NZ_BCXN01000060.1 | Matsuki,T., Yahagi,K., Mori,H., Matsumoto,H., Hara,T., Tajima,S., Ogawa,E., Kodama,H., Yamamoto,K., Yamada,T., Matsumoto,S. and Kurokawa,K.<br>https://pubmed.ncbi.nlm.nih.gov/27340092/ | Yakult Central Institute |
| *Bifidobacterium breve* | BR-14 | NZ_BCXO01000041.1 | Matsuki,T., Yahagi,K., Mori,H., Matsumoto,H., Hara,T., Tajima,S., Ogawa,E., Kodama,H., Yamamoto,K., Yamada,T., Matsumoto,S. and Kurokawa,K.<br>https://pubmed.ncbi.nlm.nih.gov/27340092/ | Yakult Central Institute |
| *Bifidobacterium breve* | BR-15 | NZ_BCXP01000023.1 | Matsuki,T., Yahagi,K., Mori,H., Matsumoto,H., Hara,T., Tajima,S., Ogawa,E., Kodama,H., Yamamoto,K., Yamada,T., Matsumoto,S. and Kurokawa,K.<br>https://pubmed.ncbi.nlm.nih.gov/27340092/ | Yakult Central Institute |
| *Bifidobacterium breve* | BR-H29 | NZ_BCXV01000018.1 | Matsuki,T., Yahagi,K., Mori,H., Matsumoto,H., Hara,T., Tajima,S., Ogawa,E., Kodama,H., Yamamoto,K., Yamada,T., Matsumoto,S. and Kurokawa,K.<br>https://pubmed.ncbi.nlm.nih.gov/27340092/ | Yakult Central Institute |

| | | | | |
|---|---|---|---|---|
| *Bifidobacterium breve* | JG_Bg463 | NZ_CABHNR010000063.1 | Hibberd C,M., Gehrig,L.J., Chang,H.-W. and Venkatesh,S.<br><br>Unpublished | The Edison Family Center of Genome Sciences and Systems Biology |
| *Bifidobacterium breve* | BR3 | NZ_CP010413.1 | Kim,J.F. and Kwak,M.-J.<br>Unpublished | Yonsei University |
| *Bifidobacterium breve* | LMC520 | NZ_CP019596.1 | Kim,S.-Y., Kang,Y.-M. and Shin,G.<br><br>Unpublished | Korea University |
| *Bifidobacterium breve* | DRBB26 | NZ_CP021390.1 | Bottacini,F., van Sinderen,D., Lambert,J., van Limpt,K. and Knol,J.<br><br>Unpublished | The Netherlands & University |
| *Bifidobacterium breve* | NRBB51 | NZ_CP021392.1 | Bottacini,F., van Sinderen,D., Lambert,J., van Limpt,K. and Knol,J.<br><br>Unpublished | The Netherlands & University |
| *Bifidobacterium breve* | M1D | NZ_JACZEL010000004.1 | Mancino,W., Mancabelli,L., Lugli,G.A., Viappiani,A., Anzalone,R.,Longhi,G., Van Sinderen,D., Ventura,M. and Turroni,F.<br><br>Unpublished | University of Parma |
| *Bifidobacterium breve* | PRL2020 | NZ_JACZEM010000004.1 | Mancino,W., Mancabelli,L., Lugli,G.A., Viappiani,A., Anzalone,R., Longhi,G., Van Sinderen,D., Ventura,M. and Turroni,F.<br><br>Unpublished | University of Parma |
| *Bifidobacterium breve* | PRL2020 | NZ_JACZEM010000005.1 | Mancino,W., Mancabelli,L., Lugli,G.A., Viappiani,A., Anzalone,R.,Longhi,G., Van Sinderen,D., Ventura,M. and Turroni,F.<br><br>Unpublished | University of Parm |
| *Bifidobacterium breve* | 1001287H _170206_ A8 | NZ_JADPBI010000004.1 | Aggarwala,V. and Faith,J.<br><br>Unpublished | Genetics and Genomic Sciences, Icahn School of Medicine at Mount Sinai |

| | | | | |
|---|---|---|---|---|
| *Bifidobacterium breve* | JCP7499 | NZ_KE993220.1 | Weinstock,G., Sodergren,E., Wylie,T., Fulton,L., Fulton,R., Fronick,C., O'Laughlin,M., Godfrey,J., Miner,T., Herter,B., Appelbaum,E., Cordes,M., Lek,S., Wollam,A., Pepin,K.H., Palsikar,V.B., Mitreva,M. and Wilson,R.K. Unpublished | Washington University School of Medicine |
| *Bifidobacterium breve* | 1 | NZ_LR655209.1 | Chang,H.-W., Raman,A., Venkatesh,S. and Gehrig,J. Unpublished | The Edison Family Center for Genome Sciences and Systems Biology |
| *Bifidobacterium breve* | 1891B | NZ_NAQG01000030.1 | Lugli,G.A., Duranti,S., Milani,C. and Mancabelli,L. Unpublished | University of Parma |
| *Bifidobacterium breve* | UMB0915 | NZ_PKGQ01000010.1 | Thomas-White,K. and Wolfe,A.J. Unpublished | Loyola University Chicago |
| *Bifidobacterium breve* | UMB0089 | NZ_PNHM01000018.1 | Thomas-White,K., Kumar,N., Forster,S., Putonti,C., Lawley,T. and Wolfe,A.J. Unpublished | Loyola University Chicago |
| *Bifidobacterium breve* | 142 | NZ_VIDR01000021.1 | Bansal,K., Sundararaman,A., Achi,S.C., Halami,P. and Patil,P.B. Unpublished | CSIR-Institute of Microbial Technology, India |

**Supplementary Table S2. Description of the genome sequences available in public database for *B. longum*, which harbour *erm*(X) and *erm*(D) genes.**

| Species | Strain | Genome Acc. No. | Authors and link | Affiliation |
|---|---|---|---|---|

| | | | | |
|---|---|---|---|---|
| *Bifidobacterium longum* | IN-07 | NZ_BCYF01000108.1 | Matsuki,T., Yahagi,K., Mori,H., Matsumoto,H., Hara,T., Tajima,S.,Ogawa,E., Kodama,H., Yamamoto,K., Yamada,T., Matsumoto,S. and Kurokawa,K.<br><br>https://pubmed.ncbi.nlm.nih.gov/27340092/ | Yakult Central Institute |
| *Bifidobacterium longum* | IN-F29 | NZ_BCYG01000035.1 | Matsuki,T., Yahagi,K., Mori,H., Matsumoto,H., Hara,T., Tajima,S.,Ogawa,E., Kodama,H., Yamamoto,K., Yamada,T., Matsumoto,S. and Kurokawa,K.<br><br>https://pubmed.ncbi.nlm.nih.gov/27340092/ | Yakult Central Institute |
| *Bifidobacterium longum* | LO-06 | NZ_BCYH01000003.1 | Matsuki,T., Yahagi,K., Mori,H., Matsumoto,H., Hara,T., Tajima,S.,Ogawa,E., Kodama,H., Yamamoto,K., Yamada,T., Matsumoto,S. and Kurokawa,K.<br><br>https://pubmed.ncbi.nlm.nih.gov/27340092/ | Yakult Central Institute |
| *Bifidobacterium longum* | LO-K29a | NZ_BCYL01000041.1 | Matsuki,T., Yahagi,K., Mori,H., Matsumoto,H., Hara,T., Tajima,S.,Ogawa,E., Kodama,H., Yamamoto,K., Yamada,T., Matsumoto,S. and Kurokawa,K.<br><br>https://pubmed.ncbi.nlm.nih.gov/27340092/ | Yakult Central Institute |
| *Bifidobacterium longum* | 2_mod | NZ_CABHMO010000006.1 | Chang,H.-W., Raman,A., Venkatesh,S. and Gehrig,J.<br><br>Unpublished | The Edison Family Center for Genome Sciences and Systems Biology |
| *Bifidobacterium longum* | JG_Bg463 | NZ_CABHNT010000033.1 | Hibberd C,M., Gehrig,L.J., Chang,H.-W. and Venkatesh,S.<br><br>Unpublished | The Edison Family Center of Genome Sciences and Systems Biology |
| *Bifidobacterium longum* | YS108R | NZ_CP029796.1 | Yan,S. and Yang,B.<br><br>Unpublished | Jiangnan University |

| | | | | |
|---|---|---|---|---|
| *Bifidobacterium longum* | LTBL16 | NZ_CP034089.1 | Huang,G.H. and Li,Q.Y.<br>Unpublished | Guangxi University |
| *Bifidobacterium longum* | ZJ1 | NZ_CP040235.1 | Jin,Z.<br>Unpublished | University of Science and Technology of China |
| *Bifidobacterium longum* | K2-21-4 | NZ_CP065395.1 | Guan,J.<br>Unpublished | Northeast Agricultrue University, China |
| *Bifidobacterium longum* | I2-2-3 | NZ_CP065397.1 | Guan,J.<br>Unpublished | Northeast Agricultrue University, China |
| *Bifidobacterium longum* | K5 | NZ_CP072500.1 | Huo,G.<br>Unpublished | Northeast Agricultrue University, China |
| *Bifidobacterium longum* | K15 | NZ_CP072501.1 | Huo,G.<br>Unpublished | Northeast Agricultrue University, China |
| *Bifidobacterium longum* | 1001302B_160321_G1 | NZ_JADMSA010000028.1 | Aggarwala,V. and Faith,J.<br>Unpublished | Genetics and Genomic Sciences, Icahn School of Medicine at Mount Sinai |
| *Bifidobacterium longum* | BSD2780061689_150309_E10 | NZ_JADNAG010000007.1 | Aggarwala,V. and Faith,J.<br>Unpublished | Genetics and Genomic Sciences, Icahn School of Medicine at Mount Sinai |
| *Bifidobacterium longum* | 1001262B_160229_E5 | NZ_JADPAC010000006.1 | Aggarwala,V. and Faith,J.<br>Unpublished | Genetics and Genomic Sciences, Icahn School of Medicine at Mount Sinai |
| *Bifidobacterium longum* | 1001254J_160919_E2 | NZ_JADPDA010000002.1 | Aggarwala,V. and Faith,J.<br>Unpublished | Genetics and Genomic Sciences, Icahn School of Medicine at Mount Sinai |

| | | | | |
|---|---|---|---|---|
| *Bifidobacterium longum* | BSD2780061687b_171204_C6 | NZ_JADPDM010000012.1 | Aggarwala,V. and Faith,J.<br><br>Unpublished | Genetics and Genomic Sciences, Icahn School of Medicine at Mount Sinai |
| *Bifidobacterium longum* | BSD2780061687b_171204_F1 | NZ_JADPFY010000014.1 | Aggarwala,V. and Faith,J.<br><br>Unpublished | Genetics and Genomic Sciences, Icahn School of Medicine at Mount Sinai |
| *Bifidobacterium longum* | C11A10B | NZ_QKOJ01000037.1 | Yan,S. Yang,B. Zhao,J. Zhao,J., Stanton,C. Ross,R.P. Zhang,H. and Chen,W.<br><br>https://pubmed.ncbi.nlm.nih.gov/30806428/ | Jiangnan University |
| *Bifidobacterium longum* | AM21-20 | NZ_QRIR01000021.1 | Zou,Y., Xue,W. and Luo,G.<br><br>Unpublished | BGI-CNGB, Yantian Dist., China |
| *Bifidobacterium longum* | AM20-39 | NZ_QRIW01000005.1 | Zou,Y., Xue,W. and Luo,G.<br><br>Unpublished | BGI-CNGB, Yantian Dist., China |
| *Bifidobacterium longum* | AM20-3 | NZ_QRIY01000031.1 | Zou,Y., Xue,W. and Luo,G.<br><br>Unpublished | BGI-CNGB, Yantian Dist., China |
| *Bifidobacterium longum* | AM20-19AC | NZ_QRIZ01000033.1 | Zou,Y., Xue,W. and Luo,G.<br><br>Unpublished | BGI-CNGB, Yantian Dist., China |
| *Bifidobacterium longum* | AM16-2 | NZ_QRKK01000014.1 | Zou,Y., Xue,W. and Luo,G.<br><br>Unpublished | BGI-CNGB, Yantian Dist., China |
| *Bifidobacterium longum* | AM12-16 | NZ_QRLQ01000020.1 | Zou,Y., Xue,W. and Luo,G.<br><br>Unpublished | BGI-CNGB, Yantian Dist., China |
| *Bifidobacterium longum* | AM10-15B | NZ_QRMG01000036.1 | Zou,Y., Xue,W. and Luo,G.<br><br>Unpublished | BGI-CNGB, Yantian Dist., China |

| | | | | |
|---|---|---|---|---|
| *Bifidobacterium longum* | AF34-9AC | NZ_QRPT01000029.1 | Zou,Y., Xue,W. and Luo,G. | BGI-CNGB, Yantian Dist., China |
| | | | Unpublished | |
| *Bifidobacterium longum* | AF26-10 | NZ_QRTY01000015.1 | Zou,Y., Xue,W. and Luo,G.<br><br>Unpublished | BGI-CNGB, Yantian Dist., China |
| *Bifidobacterium longum* | AF11-41 | NZ_QSAM01000024.1 | Zou,Y., Xue,W. and Luo,G.<br><br>Unpublished | BGI-CNGB, Yantian Dist., China |
| *Bifidobacterium longum* | AF11-12 | NZ_QSAR01000044.1 | Zou,Y., Xue,W. and Luo,G.<br><br>Unpublished | BGI-CNGB, Yantian Dist., China |
| *Bifidobacterium longum* | AM30-9LB | NZ_QSJH01000024.1 | Zou,Y., Xue,W. and Luo,G.<br><br>Unpublished | BGI-CNGB, Yantian Dist., China |
| *Bifidobacterium longum* | TF07-34 | NZ_QSRP01000023.1 | Zou,Y., Xue,W. and Luo,G.<br><br>Unpublished | BGI-CNGB, Yantian Dist., China |
| *Bifidobacterium longum* | TF07-31 | NZ_QSRQ01000019.1 | Zou,Y., Xue,W. and Luo,G.<br><br>Unpublished | BGI-CNGB, Yantian Dist., China |
| *Bifidobacterium longum* | TF01-22 | NZ_QSSW01000011.1 | Zou,Y., Xue,W. and Luo,G.<br><br>Unpublished | BGI-CNGB, Yantian Dist., China |
| *Bifidobacterium longum* | MCC10003 | NZ_SHPN01000067.1 | Odamaki,T., Bottacini,F., Kato,K., Mitsuyama,E., Yoshida,K., Horigome,A., Xiao,J.Z. and van Sinderen,D.<br><br>https://pubmed.ncbi.nlm.nih.gov/29311585/ | Morinaga Milk Industry |
| *Bifidobacterium longum* | MCC10004 | NZ_SHPO01000046.1 | Odamaki,T., Bottacini,F., Kato,K., Mitsuyama,E., Yoshida,K., Horigome,A., Xiao,J.Z. and van Sinderen,D.<br><br>https://pubmed.ncbi.nlm.nih.gov/29311585/ | Morinaga Milk Industry |

| | | | | |
|---|---|---|---|---|
| *Bifidobacterium longum* | MCC10006 | NZ_SHPP01000072.1 | Odamaki,T., Bottacini,F., Kato,K., Mitsuyama,E., Yoshida,K., Horigome,A., Xiao,J.Z. and van Sinderen,D.<br><br>https://pubmed.ncbi.nlm.nih.gov/29311585/ | Morinaga Milk Industry |
| *Bifidobacterium longum* | MCC10007 | NZ_SHPQ01000047.1 | Odamaki,T., Bottacini,F., Kato,K., Mitsuyama,E., Yoshida,K., Horigome,A., Xiao,J.Z. and van Sinderen,D.<br><br>https://pubmed.ncbi.nlm.nih.gov/29311585/ | Morinaga Milk Industry |
| *Bifidobacterium longum* | MCC10008 | NZ_SHPR01000078.1 | Odamaki,T., Bottacini,F., Kato,K., Mitsuyama,E., Yoshida,K., Horigome,A., Xiao,J.Z. and van Sinderen,D.<br><br>https://pubmed.ncbi.nlm.nih.gov/29311585/ | Morinaga Milk Industry |
| *Bifidobacterium longum* | MCC10008 | NZ_SHPR01000079.1 | Odamaki,T., Bottacini,F., Kato,K., Mitsuyama,E., Yoshida,K., Horigome,A., Xiao,J.Z. and van Sinderen,D.<br><br>https://pubmed.ncbi.nlm.nih.gov/29311585/ | Morinaga Milk Industry |
| *Bifidobacterium longum* | MCC10009 | NZ_SHPS01000026.1 | Odamaki,T., Bottacini,F., Kato,K., Mitsuyama,E., Yoshida,K., Horigome,A., Xiao,J.Z. and van Sinderen,D.<br><br>https://pubmed.ncbi.nlm.nih.gov/29311585/ | Morinaga Milk Industry |
| *Bifidobacterium longum* | MCC10010 | NZ_SHPT01000078.1 | Odamaki,T., Bottacini,F., Kato,K., Mitsuyama,E., Yoshida,K., Horigome,A., Xiao,J.Z. and van Sinderen,D.<br><br>https://pubmed.ncbi.nlm.nih.gov/29311585/ | Morinaga Milk Industry |
| *Bifidobacterium longum* | MCC10011 | NZ_SHPU01000015.1 | Odamaki,T., Bottacini,F., Kato,K., Mitsuyama,E., Yoshida,K., Horigome,A., Xiao,J.Z. and van Sinderen,D.<br><br>https://pubmed.ncbi.nlm.nih.gov/29311585/ | Morinaga Milk Industry |
| *Bifidobacterium longum* | MCC10015 | NZ_SHPX01000072.1 | Odamaki,T., Bottacini,F., Kato,K., Mitsuyama,E., Yoshida,K., Horigome,A., Xiao,J.Z. and van Sinderen,D.<br><br>https://pubmed.ncbi.nlm.nih.gov/29311585/ | Morinaga Milk Industry |

| | | | | |
|---|---|---|---|---|
| *Bifidobacterium longum* | MCC10041 | NZ_SHQS01000046.1 | Odamaki,T., Bottacini,F., Kato,K., Mitsuyama,E., Yoshida,K., Horigome,A., Xiao,J.Z. and van Sinderen,D.<br><br>https://pubmed.ncbi.nlm.nih.gov/29311585/ | Morinaga Milk Industry |
| *Bifidobacterium longum* | MCC10044 | NZ_SHQV01000041.1 | Odamaki,T., Bottacini,F., Kato,K., Mitsuyama,E., Yoshida,K., Horigome,A., Xiao,J.Z. and van Sinderen,D.<br><br>https://pubmed.ncbi.nlm.nih.gov/29311585/ | Morinaga Milk Industry |
| *Bifidobacterium longum* | MCC10052 | NZ_SHRC01000065.1 | Odamaki,T., Bottacini,F., Kato,K., Mitsuyama,E., Yoshida,K., Horigome,A., Xiao,J.Z. and van Sinderen,D.<br><br>https://pubmed.ncbi.nlm.nih.gov/29311585/ | Morinaga Milk Industry |
| *Bifidobacterium longum* | MCC10053 | NZ_SHRD01000034.1 | Odamaki,T., Bottacini,F., Kato,K., Mitsuyama,E., Yoshida,K., Horigome,A., Xiao,J.Z. and van Sinderen,D.<br><br>https://pubmed.ncbi.nlm.nih.gov/29311585/ | Morinaga Milk Industry |
| *Bifidobacterium longum* | MCC10075 | NZ_SHRW01000046.1 | Odamaki,T., Bottacini,F., Kato,K., Mitsuyama,E., Yoshida,K., Horigome,A., Xiao,J.Z. and van Sinderen,D.<br><br>https://pubmed.ncbi.nlm.nih.gov/29311585/ | Morinaga Milk Industry |
| *Bifidobacterium longum* | MCC10076 | NZ_SHRX01000044.1 | Odamaki,T., Bottacini,F., Kato,K., Mitsuyama,E., Yoshida,K., Horigome,A., Xiao,J.Z. and van Sinderen,D.<br><br>https://pubmed.ncbi.nlm.nih.gov/29311585/ | Morinaga Milk Industry |
| *Bifidobacterium longum* | MCC10096 | NZ_SHSP01000027.1 | Odamaki,T., Bottacini,F., Kato,K., Mitsuyama,E., Yoshida,K., Horigome,A., Xiao,J.Z. and van Sinderen,D.<br><br>https://pubmed.ncbi.nlm.nih.gov/29311585/ | Morinaga Milk Industry |
| *Bifidobacterium longum* | MCC10106 | NZ_SHSX01000061.1 | Odamaki,T., Bottacini,F., Kato,K., Mitsuyama,E., Yoshida,K., Horigome,A., Xiao,J.Z. and van Sinderen,D.<br><br>https://pubmed.ncbi.nlm.nih.gov/29311585/ | Morinaga Milk Industry |

| | | | | |
|---|---|---|---|---|
| *Bifidobacterium longum* | MCC10107 | NZ_SHSY01000033.1 | Odamaki,T., Bottacini,F., Kato,K., Mitsuyama,E., Yoshida,K., Horigome,A., Xiao,J.Z. and van Sinderen,D. https://pubmed.ncbi.nlm.nih.gov/29311585/ | Morinaga Milk Industry |
| *Bifidobacterium longum* | MCC10114 | NZ_SHTD01000056.1 | Odamaki,T., Bottacini,F., Kato,K., Mitsuyama,E., Yoshida,K., Horigome,A., Xiao,J.Z. and van Sinderen,D. https://pubmed.ncbi.nlm.nih.gov/29311585/ | Morinaga Milk Industry |
| *Bifidobacterium longum* | MCC10119 | NZ_SHTI01000003.1 | Odamaki,T., Bottacini,F., Kato,K., Mitsuyama,E., Yoshida,K., Horigome,A., Xiao,J.Z. and van Sinderen,D. https://pubmed.ncbi.nlm.nih.gov/29311585/ | Morinaga Milk Industry |
| *Bifidobacterium longum* | MCC10121 | NZ_SHTJ01000022.1 | Odamaki,T., Bottacini,F., Kato,K., Mitsuyama,E., Yoshida,K., Horigome,A., Xiao,J.Z. and van Sinderen,D. https://pubmed.ncbi.nlm.nih.gov/29311585/ | Morinaga Milk Industry |
| *Bifidobacterium longum* | MCC10124 | NZ_SHTL01000046.1 | Odamaki,T., Bottacini,F., Kato,K., Mitsuyama,E., Yoshida,K., Horigome,A., Xiao,J.Z. and van Sinderen,D. https://pubmed.ncbi.nlm.nih.gov/29311585/ | Morinaga Milk Industry |
| *Bifidobacterium longum* | MCC10126 | NZ_SHTN01000065.1 | Odamaki,T., Bottacini,F., Kato,K., Mitsuyama,E., Yoshida,K.,Horigome,A., Xiao,J.Z. and van Sinderen,D. https://pubmed.ncbi.nlm.nih.gov/29311585/ | Morinaga Milk Industry |
| *Bifidobacterium longum* | MCC10120 | NZ_SHTU01000043.1 | Odamaki,T., Bottacini,F., Kato,K., Mitsuyama,E., Yoshida,K.,Horigome,A., Xiao,J.Z. and van Sinderen,D. https://pubmed.ncbi.nlm.nih.gov/29311585/ | Morinaga Milk Industry |
| *Bifidobacterium longum* | UBBI-01 | NZ_SSWL01000034.1 | Sulthana,A., Lakshmi,S.G. and Madempudi,R.S. Unpublished | Centre for Research and Development, India |
